# Captive chimpanzee takes down a drone: tool use toward a flying object

**DOI:** 10.1007/s10329-015-0482-2

**Published:** 2015-09-03

**Authors:** Jan A. R. A. M. van Hooff, Bas Lukkenaar

**Affiliations:** Royal Burgers Zoo, Antoon van Hooffplein 1, 6816 SH Arnhem, The Netherlands

**Keywords:** Chimpanzee, Tool use, Drone, Weapon use

## Abstract

On 10 April 2015, a Dutch TV crew was filming at the Royal Burgers Zoo in Arnhem, The Netherlands. It was the intention to film the chimpanzees in the enclosure from close-by and from above with the means of a drone. When the drone came a bit closer to the chimpanzees, a female individual made two sweeps with a branch that she held in one hand. The second one was successful and downed the drone. The use of the stick in this context was a unique action. It seemed deliberate given the decision to collect it and carry it to a place where the drone might be attacked. This episode adds to the indications that chimpanzees engage in forward planning of tool-use acts.

**Electronic supplementary material:**

The online version of this article (doi:10.1007/s10329-015-0482-2) contains supplementary material, which is available to authorized users.

## Introduction

On 10 April 2015, video recordings were made at the Royal Burgers Zoo in Arnhem, The Netherlands, for a documentary. The filming was done with a camera-equipped drone to obtain aerial scenes of the animals and their enclosures. Also the chimpanzees were to be filmed from unusual angles. This inadvertently resulted in a remarkable example of tool use by one of the chimpanzees housed in this zoo colony, namely the use of a stick to “attack” and take down the drone.

Spontaneous and adaptive use of tools, in particular sticks, is abundant in this colony. Sticks of different sizes and shapes, logs, stones, etc., occur naturally in the area. Different artificial objects, such as boxes and rags, are introduced into the area occasionally. There has never been any explicit teaching of tool use, although the animals have had ample opportunity to watch humans handling all kind of implements.

Takeshita and van Hooff (2001) identified 13 types of tool use in this colony. The animals appeared to choose the size, shape, and weight of the tools with a particular use in mind. The tools are applied flexibly in a variety of ways. Thus, suitable objects are used as bowls, ladles, and cups to scoop up and carry water. Appropriate beams, trunks or logs are set up as stools or erected as ladders to get to places out of reach. Slender, long branches are chosen to rake in objects floating in the moat that surrounds the colony’s island. These are also used to flail fresh leaves from overhead branches of living trees protected by electrified wire from chimpanzees attempting to climb them. Short, sturdier sticks are thrown straight upward with force to hit loose fresh foliage from overhanging branches. Heavy pieces of wood and stones are chosen as throwing weapons, as happened, for instance, during an experiment in which a stuffed lion was suddenly revealed on the outer side of the moat surrounding the field. This has been nicely documented in Bert Haanstra’s (1984) documentary film about the Arnhem Zoo colony, “*The Family of Chimps*,” which also shows various other flexible uses of tools in this colony.

## Methods

Royal Burgers Zoo in Arnhem, The Netherlands, is known for its chimpanzee community, which was founded in 1971. At the time it was noteworthy because it was a successful attempt to keep a social group of considerable size and near to natural composition in captivity. The colony lives in a spacious enclosure of about 0.7 ha surrounded by a water-filled moat. It is a sloped field, partly sandy, partly grassy, with shrubs and large trees. The latter are protected against the destructive habits of the chimpanzees by electrified wire. However, a small number of trees had been sacrificed to the chimpanzees’ appetite for fresh green leaves and bark. These trees had gradually died and have been integrated into high climbing scaffolding with ropes and swings. Descriptions of the area and the management can be found in van Hooff (1973) and Adang et al. (1987). This multimale multifemale colony has become renowned for the studies of social behavior that have been conducted there, e.g., on “political” scheming and coalition formation, especially of the adult males (de Waal, 1982).

It was the camera crew’s intention to film the enclosure and the chimpanzees from close-by and from above by means of a drone-mounted camera. The recordings were meant to be used for a public relations (PR) documentary. The episode was not set up as a scientific experiment. There was no systematic behavior recording during the trial with the drone. The team present on the occasion shared what they saw from their memory immediately afterwards. The information was gathered by a zoo official who supervised the event, the second author Bas Lukkenaar.

The stick that was used to hit the drone was a willow twig of about 180 cm length. There are often willow twigs lying around. These are remnants of willow branches that have been given to the chimpanzees as a feeding pastime. The chimpanzees like to peel off the bark and eat the soft inner lining. The remnants of the branches are not removed after they have been stripped of their bark but are left in the field to play with.

## Results and discussion

A trial run was made without recordings being made. When the drone took off from the ground and made some maneuvers near and over the area, its visual appearance and humming noise caused some excitement initially. At this stage all chimpanzees were still on the ground. Some were seen to grab a willow branch, and four of them were seen to climb the scaffolding on the side where the drone was hovering, holding a branch. At this stage the significance of what was happening was not obvious to the team.

**Fig. 1:**
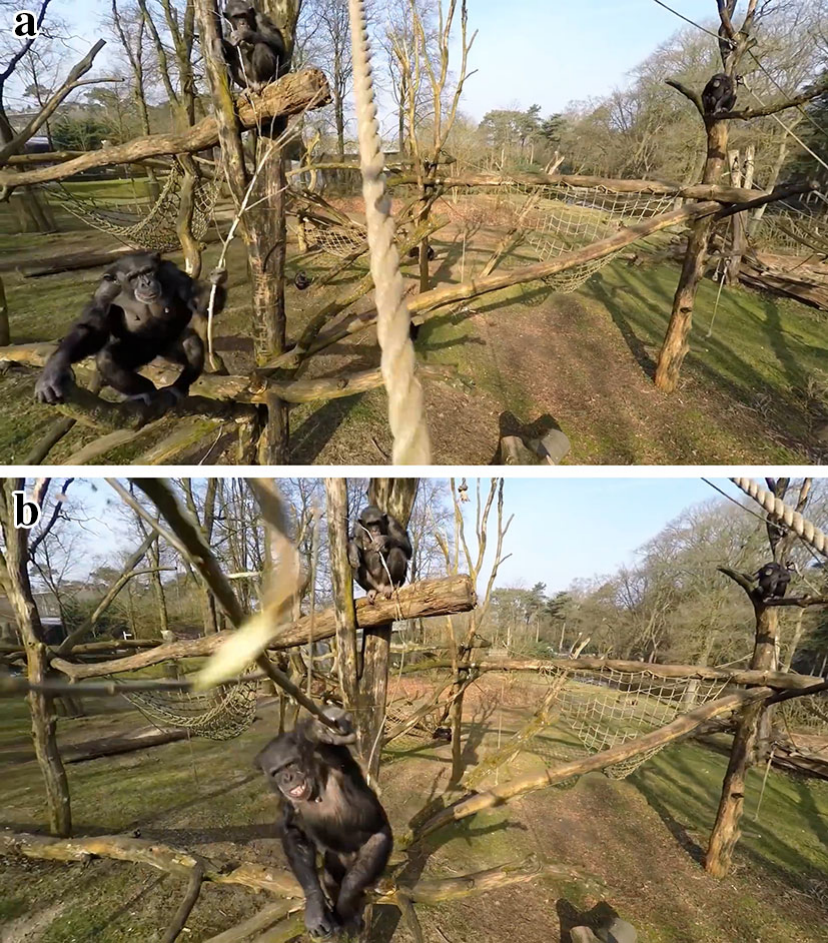
A female chimpanzee named Tushi used a stick to “attack” the drone. Behind her Raimee is also sitting with a long stick

Then a flight was made with the camera live. This flight started from outside the enclosure to make a survey shot of the area. The camera drone slowly entered the “airspace” above the apes at a height of ten to fifteen meters. By this time the chimpanzees were quiet again. Subsequently the operator closed in on two individuals that had settled at a height of about 5 m on the side of the scaffolding where the drone was and had been. These were the female Tushi, born in the colony in 1992, and the female Raimee, born in 1999. Tushi moved towards the end of a beam in the direction of the hovering drone. The operator of the drone had clearly underestimated the significance of the fact that both individuals had carried with them a long twig when they climbed the scaffolding. This is not a frequently observed behavior of these chimpanzees. When the drone came close, Tushi made two long sweeps with her branch, which she held in her left hand. The second one was successful and brought the drone down (Fig. 1). The drone was broken, but the camera continued filming. Apart from subsequent lengthy and motionless exposures of the sky and overhead branches, the camera also caught some footage of inquisitive faces of chimpanzees as they inspected and moved this strange contraption (see: https://www.youtube.com/watch?v=Z_zw8h4epQM; Online Resource 1). Initially the apes approached the motionless drone with caution, touching and moving it using short sticks. There followed some handling, dragging, and throwing about of the object, after which all of the chimpanzees lost interest. The identity of the individuals that handled the drone was not established, because by then the team was caught up with the measures taken to rescue the drone.

The sequence of events is highly suggestive of an interpretation of the use of the stick as a planned, deliberate action to “attack” the drone (agonistically motivated) or “find out about” the drone (curiosity motivated), given the decision to collect the stick and take it to a place where the drone might come within reach. However, another explanation cannot be fully excluded, i.e., that the chimpanzees grabbed a stick in a defensive “reflex” when the drone appeared. They then accidentally kept it when they climbed the scaffolding. Subsequently one individual, Tushi, found herself in a situation where the proximity of the drone released once more a defensive reflex to lash out. This two-stage reflex explanation may be cognitively more parsimonious, but it certainly is not simpler. The fact that Tushi moved towards an exposed position on the side where the drone was hovering and stayed there favors the first interpretation, as does her facial expression. There is a momentaneous grimace just before and during the act of striking. The face is tense, the teeth are bared, but there is no retraction of the mouth corners as in a “fear” face, which would have suggested that it is an agonistically motivated reflex. The precise coincidence of the facial grimace with the strike suggests that it is a concomitant of an assertive and determined exertion of force, homologous to what humans do in comparable situations.

Both in the wild and in (semi)captive settings, chimpanzees regularly use tools in a variety of motivationally and functionally different contexts (e.g., McGrew 1992, 2004; Boesch 2013). Thus, they regularly incorporate branches into their intimidation displays. Also, use of sticks or clubs as handheld weapons or projectiles has been reported from the wild (e.g., Goodall 1986). In early and remarkable experiments conducted by Adriaan Kortlandt in the 1970s (for a film see Kortlandt 1993), he confronted a group of chimpanzees with a stuffed leopard that was made to suddenly appear from under a hide. The chimpanzees spontaneously took large pieces of wood that Kortlandt had thrown around in the area beforehand and used them as clubs when attacking and destroying the leopard. A similar experiment, done at Arnhem Zoo, was filmed by Haanstra (1984). Boesch (1995) mentioned the use of clubs as weapons also in the natural situation against a live leopard. The adaptation of sticks and their use as spears by West African chimpanzees to hunt galagos is also remarkable (Pruetz and Bertolani 2007). However, despite a superficial resemblance to the former behaviors, the motivational and functional context of this form of tool use is different from that of weapon use. It is not motivated by agonistic tendencies. It is a method to procure food and as such is comparable to the use of twigs or sticks in capturing termites or ants.

Tool use of primates, especially chimpanzees (McGrew 2004) and orangutans (van Schaik et al. 2003), is both variable and often population specific. A similar variety is found in the Arnhem captive chimpanzee population (Takeshita and van Hooff 2001). Some tool-use behaviors recorded there have rarely if at all been seen in the wild. Arnhem chimpanzees may deter or tease others by throwing handfuls of loose sand in their face (Adang 1984). This habit was popular in the 1980s, but has subsequently all but died out. Another example is the use of rags in a solitary game resembling blind man’s buff and during a playful “peek-a-boo” interaction in which one individual hides its face under a towel. At Arnhem Zoo, both the necessity (the wish to get to otherwise unaccessible places) and the “ecological” opportunities (both the availability of incentives and of suitable objects) clearly influence the development of these behaviors (cf. Koops et al. 2014). These observations and the present episode add to the growing evidence about the cultural flexibility that chimpanzees show and their ability to engage in forward planning of specific acts and general activities (e.g., Janmaat et al. 2014; Osvath 2009; Mulcahy and Call 2006).

## Electronic supplementary material

Supplementary material 1 (WMV 8788 kb)
